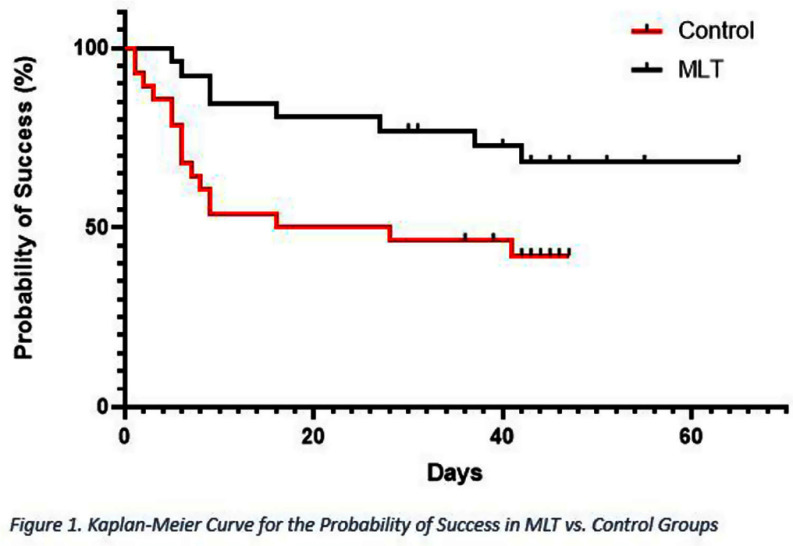# Multi-Center, Randomized Study To Evaluate The Efficacy And Safety of Mino-Lok for the management of CLABSI In Hemodialysis Patients

**DOI:** 10.1017/ash.2025.288

**Published:** 2025-09-24

**Authors:** Vinan Rathore, Anne-Marie Chaftari, Ray Hachem, Mark Rupp, Leonard Mermel, Myron Czuczman, Alan Lader, Issam Raad

**Affiliations:** 1 The University of Texas MD Anderson Cancer Center; 2 University of Nebraska Medical Center; 3 MD Anderson Cancer Center

## Abstract

**Background:** Catheter-related or central line-associated bloodstream infection (CRBSI/CLABSI) is a common and serious complication in patients undergoing hemodialysis (HD), often resulting in significant morbidity and mortality. Managing CRBSI/CLABSI often requires removing the central venous catheter (CVC) and placing a new one at a different vascular site. However, this approach is not always feasible for these patients that often have limited vascular access. No adjunct antimicrobial lock therapy has been FDA-approved for managing such infections and is urgently needed to salvage HD vascular access. Our study evaluated a novel triple combination antimicrobial catheter lock solution containing minocycline, EDTA, and ethanol (Mino Lok (MLT)). MLT has shown broad-spectrum in-vitro activity and positive results in a Phase 2 trial. Herein, we report the results of MLT CVC-salvage therapy in the subgroup of HD subjects from a phase 3 trial. **Methods:** This international, multicenter, superiority trial was conducted at 34 sites. HD, cancer, or other patients requiring a long-term CVC (LTCVC), aged ≥ 12 years, with CLABSI/CRBSI, were enrolled and randomized (1:1 ratio) to receive MLT or site-specific standard of care (SOC) antimicrobial lock therapy for 2 hours/day for 7 days. The primary endpoint was median time to catheter failure (i.e., mortality, catheter removal due to inability to administer lock or infectious-related reasons, worsening signs/symptoms, persistent or recurrent bloodstream infection, or deep-seated infection). **Results:** From February 2018 to February 2024, 54 HD patients were enrolled and randomized: 26 to MLT and 28 to SOC. Gram-negative bacteria accounted for 50% of CLABSI/CRBSIs, gram-positive bacteria 43%, and Candida species 7%. Highly virulent organisms (non-commensals) caused 69% of all cases. Patients in SOC had a significantly shorter time to catheter failure compared to MLT (p=0.03) with 25% of CVCs failing by day 6 and 50% by day 22 in SOC compared to 25% failing by day 37 in MLT (Figure 1). Similarly, 16 subjects (57%) in SOC had a CVC failure event compared to only 8 (31%) in MLT. Adverse events (AEs) and serious AEs (SAEs) were comparable between the two groups. There were no drug-related SAEs. **Conclusion:** This phase 3 pivotal study demonstrated MLT to be highly effective and superior to SOC antimicrobial lock therapy in salvaging LTCVCs associated with CRBSI/CLABSI in HD patients. MLT has broad-spectrum activity, was well-tolerated, and was not associated with drug-related SAEs. MLT may satisfy an urgent unmet need in salvaging HD catheters in patients with CRBSIs/CLABSIs.

**Figure:**